# Primary adrenal lymphoma and its mimics: clinico-radiological differential diagnosis

**DOI:** 10.3389/fendo.2025.1639878

**Published:** 2025-10-02

**Authors:** Przemysław Kłosowski, Natalia Brzezińska, Piotr Kmieć, Dominika Okroj, Sonia Zembrzuska, Mariusz Kujawa, Anna Babińska, Renata Świątkowska-Stodulska

**Affiliations:** ^1^ Department of Endocrinology and Internal Medicine, University Clinical Centre, Gdańsk, Poland; ^2^ Department of Endocrinology and Internal Medicine, Faculty of Medicine, Medical University of Gdańsk, Gdańsk, Poland; ^3^ Faculty of Medicine, Medical University of Gdańsk, Gdańsk, Poland; ^4^ Department of Radiology, Medical University of Gdańsk, Gdańsk, Poland

**Keywords:** primary adrenal lymphoma, pheochromocytoma, atypical adrenal adenoma, adrenocortical carcinoma, diffuse large B-cell lymphoma, adrenal tumor

## Abstract

**Purpose:**

Primary adrenal lymphoma (PAL) is a rare malignancy with limited data on its characteristics. It can pose diagnostic challenges in differentiating it from other adrenal masses, such as atypical adrenal adenoma (ADE), pheochromocytoma (PCC), and adrenocortical carcinoma (ACC). This study aimed to characterize patients with PAL and compare their clinical, hormonal, and radiological features with other primary adrenal lesions whose computed tomography characteristics do not match those of a typical adenoma.

**Methods:**

This retrospective, single-center study included four patient cohorts: PAL (n=12), ADE (n=31), PCC (n=18), and ACC (n=19), treated at a tertiary care hospital between January 2013 and January 2024.

**Results:**

The histopathological type of all PAL cases was diffuse large B-cell lymphoma (DLBCL). The median age at diagnosis was 70.5 (51–76) years. The diagnosis was more prevalent in males (sex ratio 1.4). Bilateral adrenal involvement was significantly more frequent in PAL patients. Clinical symptoms included general health deterioration (100%), weight loss (75%), abdominal pain (58%) and fever (41.7%). Laboratory assessments showed a higher prevalence of anemia in PAL patients compared to ADE and PCC patients. PAL cases exhibited elevated lactate dehydrogenase (LDH) and β2-microglobulin as well as lipid profile abnormalities. Radiologically, PAL lesions were predominantly homogeneous, with a median tumor size of 78.5 (20-100.5) mm. All lesions exhibited an attenuation value > 20 Hounsfield Unit (HU) and lacked calcifications. Malignant lymph node involvement was significantly more frequent in the PAL than other cohorts.

**Conclusion:**

PAL should be included in the differential diagnosis of adrenal masses, particularly in cases of bilateral involvement. This study offers insights into its clinical presentation and highlights distinguishing features compared to other primary adrenal malignancies.

## 1 Introduction

Primary adrenal lymphoma (PAL) is defined as a histologically confirmed lymphoma located in one or both adrenal glands, without prior history of a lymphoma nor significant involvement of other tissues at the time of diagnosis (1). It is estimated to represent less than 1% of all non-Hodgkin lymphomas (NHL), with diffuse large B-cell lymphoma (DLBCL) being the most common histological type (2). Less common types of B-cell-derived PALs include B-cell NHL, not otherwise specified (NOS) and mantle cell lymphoma (MCL) (3). In the DLBCL-type PAL, immunophenotypic analysis typically reveals expression of B-cell markers such as CD20, along with strong B-cell-lymphoma-2 (BCL2) positivity and frequent co-expression of myelocytomatosis oncogene (MYC); notably, dual MYC and BCL2 expression is observed in approximately 69% of cases (4). The etiopathogenesis of PAL remains poorly understood; existing data are inconsistent. Under normal conditions, the adrenal glands lack lymphoid tissue, which supports the hypothesis that chronic inflammatory conditions, such as autoimmune adrenalitis, may lead to lymphocytic infiltration and subsequent lymphomatous transformation (3). Conversely, the presence of quiescent hematopoietic tissue, structurally resembling myelolipoma, was also suggested as a potential origin of a PAL (2, 5–7). Other proposed predisposing factors include immunological disorders, a history of malignancy, and viral infections such as Epstein-Barr virus (EBV), human immunodeficiency virus (HIV), and JC virus (JCV) (8).

While adrenal masses are detected incidentally on imaging studies in approximately 5-6% of the general population, only 5-10% of these masses are malignant (9, 10). According to Chandraseka et al., PAL stands for 4.3% of primary adrenal malignancies, while adrenocortical carcinoma (ACC) 39.7%, neuroblastoma 10.9%, pheochromocytoma (PCC) 4.3% and sarcoma 1.3% (11, 12). Nonenhanced computed tomography (CT) is the recommended imaging modality for evaluating adrenal masses. A CT density >20 Hounsfield units (HU) demonstrates very high sensitivity for detecting adrenal malignancy, while adrenal masses with a density ≤10 HU are almost never malignant (10). A heterogeneous appearance, CT density >10 HU and size >4 cm may indicate malignancies such as ACC, PCC or metastases, but also benign tumors, including atypical adrenocortical adenoma (ADE) (10, 13). PAL lesions also exhibit high non-enhanced CT density, complicating the differential diagnosis. In contrast to other malignancies, adrenalectomy in patients with a PAL is generally not considered advantageous due to its aggressive nature characterized by frequent systemic spread (2, 5, 6, 11, 14, 15). Surgical intervention alone has shown limited survival benefits and is generally not curative [8, 16]. Furthermore, there is a risk of delaying the initiation of chemotherapy, which is the first line treatment in PAL. CHOP-based regimens (cyclophosphamide, doxorubicin, vincristine, and prednisone) are the most commonly administered, and the addition of rituximab has been shown to significantly improve clinical outcomes in patients with a PAL (16–18).

Its nonspecific clinical presentation, including B symptoms (fever >38°C of unknown origin, night sweats, or weight loss of >10% over the last six months), abdominal pain, and potential adrenal insufficiency (AI), coupled with limited research and PAL’s rarity, pose significant diagnostic and therapeutic challenges.

This study aimed to characterize patients with PAL and compare their clinical, hormonal, and radiologic features with those of patients with other adrenal lesions whose CT characteristics do not match those of a typical adenoma.

## 2 Materials and methods

This retrospective study included patients treated in the University Clinical Centre in Gdańsk between January 2013 and January 2024. The study was approved by the bioethics committee of the Medical University of Gdańsk (Approval No.: KB/108/2024).

**Figure 1:**
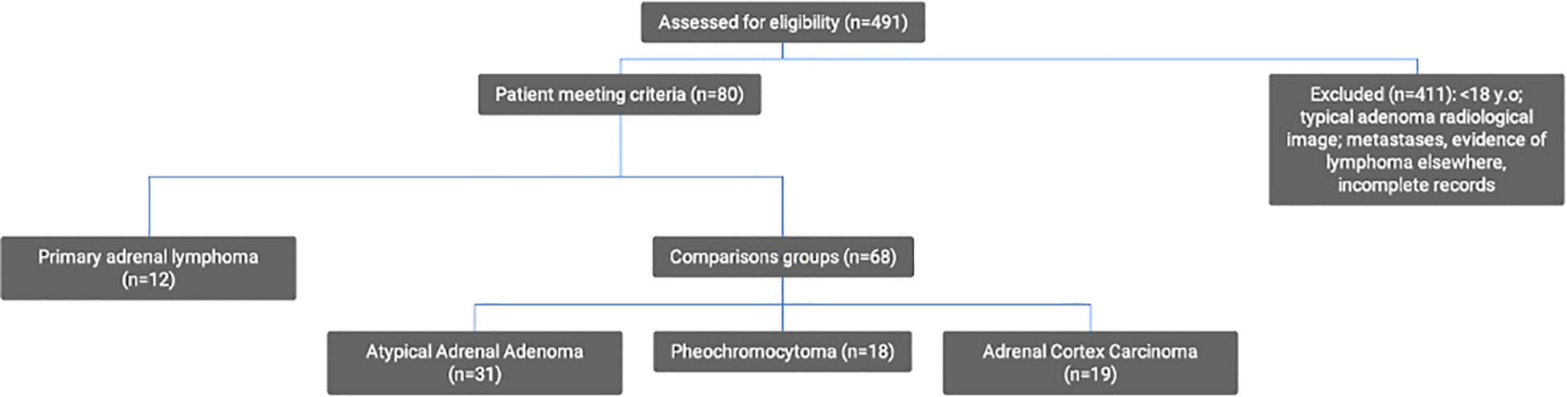
Flow chart.

Twelve consecutive patients with histologically confirmed PAL were identified from the computerized database (MedStream Design). Based on a review of the literature, ACC, PCC, ADE, angiosarcoma, metastases, and neuroblastoma were initially selected for analysis. Adrenal metastases were excluded due to the heterogeneity of this tumor group, different diagnostic approach in light of known prior primary malignancy, possible systemic spread, past therapy affecting clinical and laboratory parameters as well as in some cases distinct imaging characteristics. No cases of adrenal neuroblastoma were identified at our institution during the study period. To ensure robust statistical analysis, rare tumors were excluded so that three types were selected as cohorts for differential diagnosis: ADE, PCC, and ACC. The identification of cases was conducted by querying the same database using the terms “adrenal adenoma,” “adrenal carcinoma,” or “pheochromocytoma” and narrowing the search to those managed in the Department of Surgery of our hospital, identifying 491 patients. The following were excluded: patients below the age of 18, those with secondary adrenal involvement due to a previously diagnosed lymphoma, typical adrenal adenomas (density <10 HU), and patients lacking comprehensive medical records (histopathological examinations, radiological images, and laboratory results). After applying these criteria, 31 patients with an atypical ADE, 18 with a PCC, and 19 with an ACC were included in the study (**Figure 1**).

Radiological images, clinical characteristics, and laboratory results were analyzed. The following radiological features were considered: size, shape, density, homogeneity, presence of calcifications, and lymph node involvement. Clinical information comprised demographic data, symptoms, medical history, and physical examination. Laboratory assessments included tests conducted during hospitalizations in the endocrinology, hematology and/or emergency departments. Dehydroepiandrosterone sulfate (DHEA-S) reference ranges vary according to age and sex, whereas the measurement methods for methoxy-catecholamines changed over the course of the study. To enable direct data comparison, these parameters were expressed as a percentage of the upper limit of normal (ULN). Initially, methoxy-catecholamine levels were determined by measuring their 24-hour urinary excretion using high-performance liquid chromatography (HPLC). In 2023, this approach was replaced by a more specific plasma-based assay employing liquid chromatography–tandem mass spectrometry (LC-MS). DHEA-S concentrations were measured using immunochemiluminescence, and cortisol levels were assessed with a heterogeneous chemiluminescent immunoassay (CMIA). The reference ranges are presented in **Supplementary Tables 1, 2**.

### 2.1 Statistical analysis

Analyses were carried out using Stata 18 BE software. Shapiro-Wilk test was applied to verify the normalcy of distribution. Data were reported as number (%), average ± standard deviation (SD), or median (interquartile range, IQR), depending on variable type and distribution.

Cortisolemia in the overnight 1 mg dexamethasone suppression test (DST) was considered as a continuous variable as well as classified as abnormal or normal, based on the 50 nmol/L threshold (9). Similarly, methoxy-catecholamine levels were analyzed both as a continuous variable and in a binary manner, the latter indicating the proportion of cases with unequivocally abnormal results - defined as values exceeding at least twice the ULN.

Depending on data distribution of continuous variables, comparisons between two groups were tested with Student t test or Mann Whitney U test, and between four groups using analysis of variance (with Tukey’s *post hoc* test) or Kruskall-Wallis test (with Dunn’s *post hoc* test with Bonferroni correction). Binomial variables were analyzed with: Fisher’s exact test in the case of two groups, and Cochran’s q test with pairwise Fisher’s test with Bonferroni-Holm adjustment when four groups were compared. Presence of a PAL as a binary variable was considered in simple logistic regression. Multivariate logistic regression was considered inappropriate due to small sample size, imbalance between the number of patients with and without a PAL, variables with perfect prediction for the presence of PAL in a simple logistic regression, incomplete laboratory data for parameters previously associated with the diagnosis of a PAL and outliers in post-estimation analyses of attempted models (7). Significance was set at <0.05.

## 3 Results

### 3.1 Primary adrenal lymphoma

#### 3.1.1 General characteristics

Among the 12 PAL patients, seven were male (58%) yielding a male-to-female ratio of 1.4. The age at diagnosis ranged from 46 to 83 years, with a median age of 70.5 (51–76) years. The median body mass index (BMI) was 26.55 (22.8-27.88) kg/m². No patient had a history of an autoimmune disease or cancer at the time of PAL diagnosis. HIV and EBV antibodies were tested in seven patients, and none of them were positive.

At diagnosis, the most common symptoms were malaise (100%), followed by weight loss (75%), abdominal pain (58%), fever (42%), and night sweats (25%). Hepatosplenomegaly was present in three patients (25%) and peripheral lymphadenopathy in two patients (17%). A comprehensive summary of clinical characteristics is provided in **Table 1**. Bilateral adrenal gland involvement was observed in 7 patients (58%), all of whom underwent testing for AI by measuring cortisol, ACTH and electrolytes levels. AI was confirmed in three cases by the ACTH stimulation test, adrenal crisis (manifesting as hypotension, nausea and hyponatremia) was the initial presentation in two.

**Table 1:** Demographic and clinical characteristics of patients with PAL, ADE, PCC and ACC.

Feature	PAL	ADE	PCC	ACC	P (4 gr.)	PAL vs ADE (p)	PAL vs PCC (p)	PAL vs ACC (p)
n	12	31	18	19	-	-	-	-
Age (years)	70.5 (61–76)	65 (51.5-67)	58 (43–66)	53 (41.5–67.5)	0.1	-	-	-
Female/male ratio (%)	5/7 (41.7)/(58.3)	26/5 (83.9)/(16.1)	12/6 (66.7)/(33.3)	14/5 (73.7)/(26.3)	0.059	–	–	–
BMI (kg/m²)	26.55 (22.2–27.88)	28.7 (25.75–30.5)	25.4 (23.5–27.68)	29.7 (25.2–32.3)	0.015	0.182	0.99	0.234
Smoker (%)	4 (33.3)	2 (6.5)	2 (9.0)	9 (47.4)	0.185	–	–	–
Diabetes mellitus (%)	2 (16.7)	12 (38.7)	3 (16.7)	5 (26.3)	0.349	-	-	-
Hypertension (%)	3 (25)	23 (74.2)	10 (55.6)	12 (63.2)	0.031	–	–	–
Malaise (%)	12 (100)	3 (9.7)	0 (0)	0 (0)	<0.001	<0.001	<0.001	<0.001
Fever (%)	5 (41.7)	1 (3.2)	1 (5.6)	1 (5.3)	<0.001	<0.001	0.006	0.022
Night sweats (%)	3 (25)	1 (3.2)	1 (5.6)	4 (21.1)	0.06	-	-	-
Abdominal pain (%)	7 (58.3)	1 (3.2)	5 (27.8)	6 (31.6)	<0.001	<0.001	0.063	0.262
Weight loss (%)	1 (8.3)	3 (9.7)	4 (22.2)	7 (36.8)	<0.001	<0.001	0.008	0.066

BMI, body mass index.

#### 3.1.2 Laboratory findings

Lactate dehydrogenase (LDH) activity was elevated in nine of 12 patients (75%). β2-microglobulin was elevated in nine of 11 patients tested (82%). Inflammatory markers were elevated in most patients with a PAL: C-reactive protein (CRP) was increased in all cases; ferritin was elevated in five of seven patients (71%) and erythrocyte sedimentation rate (ESR) in three of 10 patients (30%). Lipid profile abnormalities were also observed: elevated triglycerides were found in 4 of 8 patients (50%) and decreased high-density lipoprotein (HDL) cholesterol was observed in 6 of 7 patients (86%). Epithelial oncological markers were assessed in selected patients across all groups; all results were within normal limits. A comprehensive summary of laboratory findings for PAL patients is provided in **Table 2**.

**Table 2:** Selected laboratory features of patients with PAL, ADE, PCC and ACC.

Parameter	PAL	ADE	PCC	ACC	P (4 gr.)	PAL vs ADE (p)	PAL vs PCC (p)	PAL vs ACC (p)
n	12	31	18	19	-	-	-	-
Hemoglobin (g/dl)	11.3 (± 2.4)	13.7 (± 1.8)	13.5 (± 1.4)	12.8 (± 2.4)	0.005	0.003	0.017	0.166
MCV (fl)	86.5 (± 6.5)	91.9 (± 5.7)	89.4 (± 5.4)	88 (± 5.3)	0.012	0.015	–	–
MCH (pg)	28.7 (± 2.4)	31.3 (± 1.9)	29.7 (± 2)	29.3 (± 2.6)	0.019	0.005	0.549	0.831
Leukocytes (×10^9^/l)	7.1 (4.98)	8.1 (3.1)	7.41 (3.52)	8.5 (3.84)	0.696	-	-	-
Plateles (×10^9^/l)	270 (224–297)	247 (200–296)	262 (221–297)	301 (213–354)	0.201	–	–	–
MPV (fl)	10.5 (9.9-11.1)	11 (10.6-11.5)	11 (10.5-11.5)	10.2 (9.6-10.6)	<0.001	0.071	0.206	0.775
Creatinine (mg/dl)	1.05 (0.8-1.67)	0.68 (0.6-0.8)	0.71 (0.64-0.77)	0.78 (0.6-0.97)	0.029	0.014	0.034	0.233
LDH** (U/l)	510 (237–280) n=12	- n=0	162 (150.5-173.5) n=3	475 (208.5-703) n=7	0.058	-	-	-
β2-microglobulin (mg/dl)	5.85 (3.51–7.96) n=11	1.947 n=1	1.453 n=1	2.406 (1.74-2.5) n=3	–	–	–	–
CRP (mg/l)	71.89 (36.5–121) n=12	8.77 (2–10.7.) n=14	10.61 (1.79-14.3) n=11	63.77 (2.9-103.5) n=15	<0.001	0.002	0.01	1
ESR (mm/h)	20 (14.25–34.75) n=10	2 n=1	21 (20–22) n=2	7 (4.5-9.5) n=2	–	–	–	–
Ferritin (ng/ml)	1274 (431-2021) n=7	147 (93-147), n=6	-, n=0	86 (68-103), n=3	0.003	0.01	-	0.005
TAG (mg/dl)	150 (97.5–172) n=8	137 (80.5–162) n=11	54 n=1	161 (161–169) n=5	0.235	–	–	–
HDL cholesterol (mg/dl)	27 (17.3–39.8) n=7	64 (35–65.5) n=11	86 (71.5–105.5) n=2	42 (28–42) n=5	0.179	-	-	-
Albumin (g/l)	34.5 (28–35.5) n=12	36 (36–36) n=1	38 (36–43) n=7	34.5 (25.5–39.5) n=12	0.087	–	–	–
Corrected calcium (mg/dl)	9.69 (9.32–10.4) n=12	9.5 (8.9–9.9) n=1	9.66 (9.4–9.8) n=7	9.42 (9.2–9.82) n=6	0.46	-	-	-
Post DST cortisol (nmol/l)	142 (41.5–293) n=6	97 (70–145) n=29	14 (113.8–89) n=4	90.5 (29.5–216.2) n=12	0.36	–	–	–
Abnormal DST (%)	4 (66.7) n=6	23 (79.3) n=29	2 (66.6) n=4	7 (58.3) n=12	0.014	0.722	0.37	1
DHEA-S*** (µmol/l)	73.1 (7.5–109) n=9	21.4 (7.5–70.1) n=31	87.1 (37.2–138) n=18	187.3 (76.3–313.2) n=16	<0.001	0.843	1	0.078
DHEA-S > ULN n (%)***	0 (0) n=9	1 (3.2) n=13	6 (31.6) n=18	8 (50) n=16	<0.001	1	1	0.136
Metanephrines ULN % ***	30% (12–67) n=7	41% (27–60.5) n=27	327.4 (92–535) n=17	64.5 (39–77) n=16	<0.001	1	0.002	0.704
Normetanephrines ULN %***	89 (40–104) n=7	78 (49–111) n=27	219 (147–666) n=17	79.5 (67.5–92.5) n=16	<0.001	1	0.011	1
% patients with metanephrine/normetanephrine > 2× ULN	14.3% n=7	3.7% n=27	88.2% n=17	0 n=16	<0.001	0.374	<0.001	0.304

The number of individuals in the group (n) is provided only for outcomes that were not assessed in all participants within the group. The absence of group size information indicates that the assessment was performed for all participants.

MCV, mean corpuscular volume; MCH, mean corpuscular hemoglobin; RDW, red blood cell distribution width; MPV, mean platelet volume; LDH, lactate dehydrogenase; CRP, c-reactive protein; ESR, erythrocyte sedimentation rate; TAG, triglycerides; HDL, high-density lipoprotein; DST, dexamethasone suppression test; ULN, upper limit of normal.

**Statistical analysis was performed only between two groups due to the insufficient number of examination results in the other groups.

***Normal range depended on patient’s age, sex and the year of examination.

#### 3.1.3 Imaging

Unenhanced CT scans were available for all PAL patients, while contrast-enhanced CT in only one. Bilateral adrenal involvement was observed in seven patients (58%). In unilateral lesions, the right adrenal gland was affected in three cases, and the left in two. Median tumor diameter was 78.5 (20-100.5) mm. Median non-contrast CT density was 38.5 (34–40) HU. All lesions exhibited an attenuation value > 20 (HU). Homogeneity was noted in 11 of 12 cases (91.7%), eight tumors were oval (66.7%) and the remaining four irregularly shaped (33.3%). Calcifications were absent in all cases. Malignant regional lymph nodes were present in eight patients (66.7%). A comprehensive summary of radiological features is provided in **Table 3**.

**Table 3:** Radiological characteristics of patients with PAL, ADE, PCC and ACC.

Feature	PAL	ADE	PCC	ACC	P (4 gr.)	PAL vs ADE (p)	PAL vs PCC (p)	PAL vs ACC (p)
n	12	31	18	19	-	-	-	-
Bilateral adrenal involvement (%)	7 (58.3)	1 (3.2)	2 (11.1)	0 (0)	<0.001	0.002	0.013	0.002
Right/left side lesion (%)	3 (25)/2 (16.7)	10 (32.3)/20 (64.5)	12 (66.7)/4 (22.2)	10 (52.6)/9 (47.4)	<0.05	–	–	–
Homogeneity (%)	11 (91.7)	18 (58.1)	3 (16.7)	5 (26.3)	<0.001	0.067	<0.001	<0.001
Maximum dimension* (mm)	78.5 (20.1–105)	37 (29–47)	38.5 (25–44)	59 (44–133)	0.006	0.106	0.281	1
Density* (HU)	38.5 (34–40)	25 (16–30)	38.5 (25–44)	38.5 (25–44)	<0.001	0.004	1	1
Calcification (%)	0 (0)	4 (12.9)	1 (5.6)	6 (31.6)	<0.001	0.067	<0.001	<0.001
Irregular shape (%)	4 (33.3)	2 (7.2)	3 (16.7)	6 (31.6)	0.058	-	-	-
Pathological lymph nodes (%)	8 (66.7)	7 (22.6)	3 (15.8)	3 (15.8)	0.91	–	–	–

*In the case of bilateral adrenal lesions values of the larger and more dense lesion are given.

#### 3.1.4 Histopathological findings

Nine patients were diagnosed via CT/ultrasound-guided core needle biopsy, while in three adrenalectomy was performed. Histopathological examination revealed DLBCL in all patients. Ki67 positivity was high in all cases, at minimum 70%, on average 81 ± 9%. Bone marrow biopsy was performed in nine patients, with involvement detected in one case. Cerebrospinal fluid analysis or MRI revealed central nervous system involvement in one patient.

Immunohistochemical analysis was available in 11 out of 12 patients with PAL. All cases in this group showed positive expression of CD20, as well as high expression of BCL2 and BCL6. Whenever possible, the PAL phenotype was determined according to the Hans algorithm and MYC/BCL2 co-expression was evaluated (19). The results of these analyses are presented in **Supplementary Tables 3, 4**.

#### 3.1.5 Treatment regimens and survival outcomes

Among the 12 patients diagnosed with PAL, the most commonly used chemotherapy regimen was R-CHOP, administered either alone or in combination with other protocols such as HD-MTX (high-dose Methotrexate), R-DHAP (Rituximab, Dexamethasone, High-dose Cytarabine, Cisplatin), or R-MATRIX (Rituximab-based intensive chemotherapy protocol). Two patients additionally underwent autologous stem cell transplantation. Two patients died before treatment initiation. Survival outcomes varied, with a median survival of 12 months among patients with available follow-up data. Three patients survived beyond one year, and two remained alive over 3 and 8 years, respectively. However, follow-up information was unavailable for four cases, limiting comprehensive survival analysis. International Prognostic Index (IPI) scores were available for 11 out of 12 patients. The scores ranged from 1 to 5, with a median IPI score of 3, indicating an intermediate-to-high risk profile in most cases. Detailed information is presented in **Table 4**.

**Table 4:** Chemotherapy regimens, survival outcomes and IPI scores in patients with Primary Adrenal Lymphoma.

Patient no.	Chemotherapy regimen	Survival outcome	IPI score
1	No follow-up available	No follow-up available	3
2	8 cycles of R-CHOP	Over 8 years	1
3	8 cycles of R-CHOP, 4 cycles of HD-MTX	Over 3 years	4
4	Died before treatment	0 years	4
5	8 cycles of R-CHOP, 2 cycles of R-DHAP	16 months	3
6	7 cycles of R-CHOP, auto-HSCT (Endoxan), MTX + Depocyte (intrathecal)	Over 1 year	4
7	6 cycles of R-CHOP, auto-PBSCT	Over 23 months	3
8	No follow-up available	No follow-up available	3
9	2 cycles of R-CHOP, 1 cycle of R-miniCHOP, 3 cycles of R-CHOP with gemcitabine	10 months	5
10	Died during diagnosis	0 years	3
11	6 cycles of R-CHOP, 3 cycles of HD-MTX, R-MIV, R-MATRIX	Over 12 months	3
12	No follow-up available	No follow-up available	4

R-CHOP, Rituximab, Cyclophosphamide, Doxorubicin, Vincristine (Oncovin), Prednisone; HD-MTX, High-dose Methotrexate; R-DHAP, Rituximab, Dexamethasone, High-dose Cytarabine (Ara-C), Cisplatin; HSCT, Hematopoietic Stem Cell Transplantation; PBSCT, Peripheral Blood Stem Cell Transplantation; R-MIV, Rituximab, Methotrexate, Ifosfamide, Etoposide; R-MATRIX, Rituximab-based intensive chemotherapy protocol; IPI, International Prognostic Index.

### 3.2 Comparisons between PAL and other adrenal tumors

#### 3.2.1 Clinical characteristics

A trend toward higher median age at PAL diagnosis than for ADE, PCC, or ACC could be noticed, although the difference was not statistically significant (p=0.1), **Table 1**. PAL patients were less frequently diagnosed with hypertension compared to ADE patients (p=0.031). Prevalence of diabetes was comparable in studied groups (p=0.349).

Bilateral adrenal involvement was significantly more common in PAL patients (p<0.001), and associated with AI. Malaise and fever were more frequently observed in PAL cases than in other cohorts (p<0.001). Abdominal pain was more prevalent in patients with PAL (58.3%), PCC (27.8%), and ACC (31.6%) compared to ADE patients (0%; p<0.001). The difference in the prevalence of night sweats bordered statistical significance (p=0.06). The prevalence of weight loss was significantly higher in patients with PAL (75%) compared to those with ADE (3.2%; p<0.001), and comparable to those with PCC (22.2%; p=0.008) and ACC (36.8%; p=0.066).

#### 3.2.2 Laboratory findings

Laboratory assessments showed that PAL patients had significantly lower hemoglobin levels compared to those with ADE (p=0.003) and PCC (p=0.017), but comparable to ACC (p=0.166). A trend toward lower MCV and MCH was observed in PAL patients. RDW was significantly lower in PAL compared to ADE (p=0.013) but not significantly different from PCC (p=0.105) or ACC (p=1).

Despite comparable age and BMI, PAL patients also demonstrated higher serum creatinine levels compared to ADE (p = 0.014) and PCC (p=0.034), but no significant difference compared to ACC (p=0.233). Total white blood cell, lymphocyte, neutrocyte or monocyte counts were comparable between study groups (see more in **Supplementary Table 5**). Patients with a PAL as well as those with an ACC tended to have lower albuminemia in comparison to ADE and PCC, but the differences were not statistically significant (p=0.087). CRP was higher in patients with a PAL than those with an ADE (p=0.002) or a PCC (p=0.01).

Hormonal assessment showed comparable DST cortisol in four cohorts (p=0.102). Prevalence of abnormal DHEA-S concentrations were also comparable. Metanephrine and normetanephrine levels were significantly elevated only in PCC patients (p < 0.001). AI was observed only in the PAL cohort. Comparisons of laboratory findings between PAL and other adrenal tumors are presented in **Table 2** and **Supplementary Table 5**.

Radiologically, PAL and ADE tumors were predominantly homogeneous, whereas PCC and ACC lesions were more often heterogeneous. Tumor size differed between the cohorts, although PAL dimensions were comparable to all control groups. The PAL cohort exhibited higher unenhanced tumor densities compared to ADE (p=0.004), while values were similar to those observed in PCC and ACC. Calcifications were absent in PAL tumors but were present in 12.9% of ADE, 11.1% of PCC, and 31.6% of ACC tumors (p < 0.001). Most PAL lesions were oval in shape, with 33% showing irregular contours- similar to ADE (22.6%), PCC (16.7%), and ACC (31%) (p = 0.769). Malignant lymph nodes were significantly more frequent in the PAL group (66.7%) compared to other cohorts (p < 0.001). Radiological findings are summarized in **Table 3**.

#### 3.2.3 Simple logistic regression for the presence of PAL

Associations between the presence of PAL and other variables were tested by simple logistic regression. Odds ratios (ORs) for the investigated outcome were significantly higher for age, male sex, several clinical symptoms (fever, abdominal pain, weight loss, AI, and general worsening), homogeneity of an adrenal mass, bilateral adrenal involvement, presence of abdominal lymphadenopathy, ACTH, and anemia (**Table 5**). OR was lower in the case of hypertension and elevated DHEA-S. MCV and MCH were two continuous variables associated negatively with the presence of PAL.

**Table 5:** Simple logistic regression for the presence of PAL.

Feature/examination	Odds ratio	Standard error	P	95% confidence interval	
Age	1.1	0.02	0.042	1.00	1.11
Male sex	4.6	2.96	0.02	1.27	16.32
BMI	0.9	0.07	0.146	0.76	1.04
Smoker	0.4	0.25	0.134	0.10	1.35
Diabetes mellitus	0.5	0.39	0.37	0.1	2.39
Hypertension	0.17	0.12	0.013	0.042	0.69
General worsening	*perfect prediction (all PAL cases exhibited worsening of general condition)*				
Fever	47.9	55.77	0.001	4.88	469.68
Night sweats	3.4	2.73	0.118	0.73	16.27
Abdominal pain	8.1	5.51	0.002	2.15	30.69
Weight loss	14	10.34	<0.001	3.29	59.55
Adrenal insufficiency	*perfect prediction (only PAL cases exhibited adrenal insufficiency)*				
Bilateral lesions	22.4	17.47	<0.001	4.86	103.33
Homogenity	17.8	19.08	0.007	2.17	145.79
Maximum dimension* (mm)	1.0	0.01	0.137	1.00	1.02
Mean density* (HU)	1.1	0.037	0.065	1.00	1.14
Calcification	*perfect prediction (no PAL case exhibited calcifications)*				
Irregular shape	1.61	1.1	0.498	0.42	6.15
Pathological lymph nodes	32	25.61	<0.001	6.67	153.62
Hemoglobine	0.6	0.1	0.004	0.46	0.86
MCV	0.9	0.05	0.029	0.77	0.99
MCH	0.7	0.10	0.032	0.56	0.97
Leukocytes	0.9	0.09	0.549	0.78	1.14
Platelets	1	0.00	0.529	0.99	1.01
MPV	0.6	0.22	0.165	0.3	1.23
Creatinine	1.7	0.65	0.138	0.84	3.62
LDH	1.0	0.00	0.339	1.00	1.00
CRP	1.0	0.01	0.05	1.00	1.02
ESR	1.1	0.06	0.204	0.96	1.21
Corrected calcium	1.6	0.45	0.069	0.96	2.81
Post DST cortisol	1.0	0.00	0.552	1.00	1.01
Abnormal DST	1.2	1.12	0.816	0.21	7.34
DHEA-S	1.0	0.00	0.483	0.99	1.00
Abnormal DHEA-S	*perfect prediction (no PAL case exhibited abnormal DHEA-S concentration)*				
Normetanephrine	1.0	0.00	0.627	0.99	1.00
Metanephrine	1.0	0.01	0.262	0.96	1.01
Metanephrine/normetanephrine > 2x ULN	0.7	0.76	0.716	0.07	6.32
ACTH	1.0	0.02	0.026	1.01	1.08

BMI, body mass index; MCV, mean corpuscular volume; MCH, mean corpuscular haemoglobin; MPV, mean platelet volume; LDH, lactate dehydrogenase; CRP, c-reactive protein; ESR, erythrocyte sedimentation rate; DST, dexamethasone suppression test; ULN, upper limit of normal.

*In the case of bilateral adrenal lesions values of the larger and more dense lesion are given.

Importantly, only PAL cases exhibited AI (p=0.003 in Fisher’s exact test), and worsening of general condition was present in all PAL cases (p<0.001). Calcifications were not present in any PAL lesion.

## 4 Discussion

This study analyzed the clinical, biochemical, and radiological features of PALs in comparison with other adrenal masses with non-benign radiological presentation. PALs were generally large masses, with a median diameter of 78.5 mm (range: 17–162 mm) and a median non-contrast CT density of 38 HU (range: 25–44 HU). These findings align with previous studies, which reported median diameters of ~7–8 cm and median density of 31–37 HU (2, 7, 17, 18, 20). Unenhanced density was not helpful in differentiating between PCC and ACC. Almost all our PAL masses were homogenous, which contrasted with ACC and PCC lesions.

In our cohort, MRI and PET-CT were available only in few patients and were therefore excluded from the analysis. Literature data indicate that PALs are typically iso- or hypointense on T1, hyperintense on T2, show restricted diffusion on DWI, and demonstrate high metabolic activity on PET-CT (2, 18). In a study by Majidi et al. (17), all PAL tumors exhibited high 18FDG uptake (median SUVmax = 17), and PET-CT revealed extra-adrenal disease in 50% of patients that was missed on conventional CT. The same (high SUVmax, often >20, as well as extraadrenal involvement not detected by CT) was observed by Evangelista et al. (21), who also highlighted comparable performance of PET-CT and PET/MRI with the added benefit of superior soft-tissue contrast, as well as the value of PET-derived metrics (SUVmax, MTV) for response assessment.

A valuable complement to conventional imaging techniques may be the use of radiomics- an innovative tool that enables quantitative analysis of CT and MRI images by extracting features beyond human visual perception. In the assessment of adrenal lesions, radiomics supports the differentiation of benign and malignant tumors, particularly in diagnostically challenging cases. Tissue heterogeneity is analyzed through the extraction of first-, second-, and higher-order features, allowing for the identification of subtle differences between lipid-poor adenomas and malignant lesions such as metastases, PCC, or ACC (22). Ho et al. demonstrated that second-order textural features, such as entropy, improve diagnostic accuracy in distinguishing lipid-poor adenomas from malignant tumors (23). In PET-based radiomic studies, Wang et al. observed that specific textural features derived from PET images correlated with prognosis in patients with primary adrenal and renal lymphoma, suggesting that PET-based texture analysis may serve as a valuable non-invasive tool for risk stratification and prognostic evaluation in PAL (24). Although radiomic analysis was not feasible in our cohort at the current stage, a retrospective evaluation is planned in the near future. It will include manual or semi-automated segmentation of adrenal lesions on CT images using 3D Slicer software, followed by feature extraction according to the Imaging Biomarker Standardization Initiative (IBSI) guidelines. The extracted features- including first-order statistics and higher-order textural parameters (e.g., GLCM, GLRLM, GLSZM)- will undergo normalization and redundancy reduction. For the development of predictive models distinguishing benign from malignant lesions, machine learning algorithms such as random forest and support vector machines (SVM) will be applied. Model performance will be assessed using cross-validation techniques, with key diagnostic metrics including sensitivity, specificity, and the area under the receiver operating characteristic curve (AUC). The planned project aims to provide a robust methodological foundation for future prospective studies evaluating the utility of radiomics in adrenal lesion diagnostics, including rare entities such as PAL.

Despite characteristic radiological features, clinical presentation remains a critical component in differentiating PALs from other adrenal masses. According to published studies, only 1% to 14% of PAL cases are discovered incidentally (17, 18). In our cohort, the most commonly reported symptoms included: malaise, weight loss, abdominal pain and fever which is consistent with other studies (2). In line with our data, Rashidi et al. indicate that the prevalence of classic B symptoms was 68% among 187 lymphoma patients worldwide, while other symptoms such as anorexia, nausea/vomiting, neurological symptoms, and diarrhea were reported with lower frequencies (23%, 14%, 7%, 4%, respectively) (18). Although B-type symptoms are commonly considered nonspecific, it should be noted that worsening in general health occurred significantly more often in patients with PAL compared to our three other cohorts. A similar relationship was also demonstrated for weight loss, whereas abdominal pain was reported significantly more frequently in patients with PAL compared to those with PCC or ADE, with comparable prevalence in PAL and ACC groups.

Bilateral adrenal gland involvement in PAL varies across studies, a 58% prevalence was noted here, which is in line with a range of 33.2% to 81% in other studies (2, 11, 17, 18, 20). Interestingly, Chandrasekar et al. demonstrated bilateral adrenal involvement in only one third of 202 PAL cases but at the same time this diagnosis was the most frequent one among primary malignancies involving both glands (ACC 1.3%, PCC and paraganglioma 1.4%, neuroblastoma 1.8%, sarcoma 4.9%) (11). AI is commonly seen in patients with bilateral involvement, although it was not systematically evaluated. It was stated in 43% of bilateral cases in our study, which is lower than 61-73% prevalence rates in studies by Laurent et al., Rashidi et al. or Majidi et al. (2, 17, 18). These rates are still notably higher than those of metastatic cancers, i.e. 21-33% (25, 26). Rashidi et al. noted that AI typically develops with bilateral involvement and often follows extensive tumor replacement of adrenal parenchyma. They also highlighted that AI may present subacutely or rapidly, with nonspecific symptoms such as fatigue and hypotension. In contrast to adrenal metastases, adrenal function in PAL is more frequently compromised due to the destructive nature of the infiltration (18).

In the remainder of our PAL cohort, 1-mg DST cortisol was comparable with three other cohorts. Interestingly, Papageorgiou et al. reported cases where PAL was associated with autonomous cortisol secretion and Cushing’s syndrome. These findings were supported by immunohistochemical evidence of 17-alpha-hydroxylase expression in the lymphoma cells (27). Concerning DHEA-S, a trend was observed indicating lower levels in PAL compared to ACC patients, likely reflecting the higher prevalence of AI in the course of PAL and androgen excess in the course of ACC. Studies by Mantero et al. and Arlt et al. showed that DHEA-S concentrations are elevated in 17–42% of ACC cases, compared to just 2–3% of ADE cases (28, 29). Our results align with these findings.

Elevated inflammatory markers (CRP, ferritin), as well as increased LDH and β2-microglobulin levels, were observed in PAL patients, consistent with previous studies (2, 7, 30). In the ACC cohort, LDH was assessed in 7 out of 19 cases, with a moderately elevated median activity. This observation is in line with other studies (8, 31). Anemia is relatively common in adrenal lymphomas (3, 8). Compared to other patient groups, hemoglobin was lowest in our PAL cohort. The cause of anemia is likely multifactorial, potentially resulting from chronic inflammation, bone marrow involvement due to lymphoma, and AI.

An important yet understudied aspect of PAL are lipid profiles. Research conducted by Wang et al. demonstrated significantly reduced HDL-C levels in PAL patients compared to healthy individuals and those with other adrenal disorders (7). The authors suggest that decreased HDL-C may reflect systemic inflammation or cytokine activity associated with lymphoma progression. The diagnostic utility of HDL-C in differentiating PAL was also highlighted by Kai Yu et al., where lower HDL-C levels were identified as one of the strongest independent factors distinguishing PAL from other adrenal conditions in a multivariate analysis (14). Regrettably, in our cohorts, lipid profiles were not routinely assessed; data were available for 7/12 PAL cases, 11/33 ADE cases, 5/19 ACC cases, and only 2 PCC cases. Nevertheless, consistent with others, HDL-C was lower in the PAL cohort. Also similar to other studies, supporting the effect of inflammation on lipid profiles, CRP was high in the PAL cohort.

The study by Kai Yu et al. introduced a nomogram designed to diagnose a PAL using four key predictors: patient age, bilateral adrenal masses identified via imaging, HDL-C level, and LDH activity (14). A scoring system assigned a maximum of 19 points for advanced age, 26 points for HDL-C, 100 points for elevated LDH levels, and an additional 5 points for the presence of bilateral adrenal masses. We applied this nomogram to our cohort: six out of seven cases with all required data had a predicted probability of a PAL exceeding 95%, while in one patient the probability was only 10%, which was attributable to young age and low LDH, despite the presence of bilateral lesions. In our study, simple logistic regression revealed that the presence of PAL is associated with clinical symptoms, bilateral adrenal involvement, homogeneity of the lesion, absence of calcifications, and anemia, among others.

Specific prognostic factors in PALs have not been clearly established. Traditional risk stratification tools used in DLBCL, such as the IPI score, as well as individual parameters like LDH activity or laterality of adrenal involvement, have limited predictive value in PAL (4). While our study focused on clinical, biochemical, and radiological characteristics, emerging molecular and genetic data provide important complementary insights into the biology of PAL. In DLBCL-type PAL, immunophenotypic analysis typically demonstrates expression of B-cell markers such as CD20, with strong BCL2 positivity and frequent MYC co-expression; notably, dual expression of MYC and BCL2 is observed in approximately 69% of cases (4). This co-expression defines the so-called “double-expressor” phenotype, which is a well-established adverse prognostic factor in DLBCL and is associated with shorter survival and higher relapse risk (32). The majority of PAL cases have also been classified as the non-germinal center B-cell (non-GCB) subtype, a phenotype known to correlate with worse outcomes in DLBCL (33). Furthermore, high PD-L1 expression has been documented in selected PAL cases, raising the possibility of future immunotherapeutic approaches (34). On the genetic level, recurrent mutations in MYD88 and CD79B have been reported in PAL, with CD79B mutations in particular associated with adverse prognosis (35). Chromosomal translocations typically seen in other lymphoma subtypes, such as t(14,18) (BCL2), t(8,14) (MYC), or t(11,14) (CCND1), appear to be absent in PAL. In contrast, BCL6 gene rearrangements are frequently observed and may contribute to PAL pathogenesis, although they lack diagnostic specificity (33). Among the available immunohistochemical marker expression results in our PAL cohort, CD20 expression was observed in all patients, and a high rate of BCL2 expression was noted (positive in 10 out of 11 cases). The double expressor phenotype could be assessed in 5 patients, of whom 3 out of 5 demonstrated positive MYC/BCL2 co-expression. Similar to the findings reported by Mozos et al., the non-GCB phenotype was predominant (33). These findings emphasize the biological distinctiveness of PAL among extranodal DLBCLs and highlight the potential value of molecular profiling in guiding future diagnostic and therapeutic strategies. In our cohort, R-CHOP-based chemotherapy was the most frequently administered regimen, often combined with intensification strategies or central nervous system (CNS) prophylaxis. The study by Kim et al., which included 31 patients with adrenal DLBCL treated with a median of six R-CHOP cycles, reported 2-year overall survival (OS) and progression-free survival (PFS) rates of 68% and 51%, respectively (36). Complete and partial remission were achieved in 55% and 32% of patients, respectively, with complete remission (CR) serving as a significant predictor of improved OS and PFS (36). These findings support the use of R-CHOP as a standard first-line treatment for primary adrenal DLBCL. Although our series is limited by its smaller size, the observed trend toward prolonged survival in patients achieving CR further emphasizes the value of early and effective systemic therapy.

## 5 Strengths and limitations of the study

The limitations of this study include its retrospective and single-center design without external validation. Small sample size and an incomplete laboratory dataset for parameters previously associated with a PAL further limit the conclusions that could be drawn. Multivariate analysis was not possible due to an imbalance in the number of patients with and without a PAL, the presence of variables exhibiting perfect prediction in univariate logistic regression, and outliers observed in post-estimation diagnostics. Only unenhanced CT was included for radiological comparison between groups. Other imaging modalities, such as contrast-enhanced CT, MRI (including T1/T2 signals and DWI), and PET-CT (SUVmax), were excluded due to insufficient data. However, non-contrast CT remains often the first-line imaging modality, and radiological PAL features observed on this modality should not be overlooked.

Particularly in the context of bilateral adrenal involvement, metastases to the glands should be considered. Our study deliberately excluded these cases to maintain methodological consistency by focusing exclusively on primary adrenal tumors with overlapping radiological features rather than a widely heterogeneous group with frequent history of prior therapy of the primary malignancy. Furthermore, histological confirmation of adrenal metastases is often lacking in retrospective data, limiting their inclusion. Future studies with larger, multicenter cohorts may address this important diagnostic group.

Despite these limitations, the study incorporates clinical, biochemical, and radiological analyses of PAL, ADE, PCC, and ACC cohorts for a comprehensive differential diagnosis. To our knowledge, it is among few studies to compare these adrenal pathologies in a unified analytical framework.

## 6 Clinical implications and recommendations

Diagnosis of PAL should be considered in patients presenting with nonspecific systemic symptoms, such as significant deterioration in general condition, weight loss or fever of unknown origin. A characteristic indication for suspecting PAL is bilateral adrenal involvement observed in imaging studies, particularly when accompanied by adrenal insufficiency. Radiologically, PAL-type tumors are characterized by homogeneous lesions with increased density (>20 HU) on CT and the absence of calcifications within the tumor.

Laboratory findings that may support the diagnosis include elevated LDH and β2-microglobulin levels, normocytic anemia, and reduced HDL-C. In contrast, features more indicative of other adrenal tumors-such as elevated metanephrines in PCC or markedly increased DHEA-S in ACC- are generally absent in PAL. When clinical, radiologic, and laboratory features suggest PAL, image-guided core needle biopsy remains the definitive diagnostic modality, enabling timely initiation of appropriate therapy, such as chemotherapy.

## Data Availability

The raw data supporting the conclusions of this article will be made available by the authors, without undue reservation.
