# Supplementary material for: Primary adrenal lymphoma and its mimics: clinico-radiological differential diagnosis
Source: Front Endocrinol (Lausanne). 2025 Oct 2;16:1639878. doi: 10.3389/fendo.2025.1639878 (PMC12527894; doi:10.3389/fendo.2025.1639878)
Supplement: Supplementary file 1 [file Table1.docx]

**Supplementary material**

**Table S1. Laboratory reference ranges for methoxy-catecholamines.**

| **Parameter** | **Reference Range** | **Method** | **Year** |
| --- | --- | --- | --- |
| **Plasma metanephrine** | 0.04–0.45 nmol/L | LC-MS/MS | $\geq2023$ |
| **Plasma normetanephrine** | 0.11–0.74 nmol/L | LC-MS/MS | $\geq2023$ |
| **Plasma 3-methoxytyramine** | 0–0.11 nmol/L | LC-MS/MS | $\geq2023$ |
| **Urinary metanephrine (daily excretion)** | <320 µg/24h | HPLC (24-h urine) | <2023 |
| **Urinary normetanephrine (daily excretion)** | <390 µg/24h | HPLC (24-h urine) | <2023 |
| **Urinary 3-methoxytyramine (daily excretion)** | 103–434 µg/24h | HPLC (24-h urine) | <2023 |

*LC-MS- liquid chromatography–tandem mass spectrometry, HPLC- high-performance liquid chromatography.*

**Table S2. Laboratory reference ranges for dehydroepiandrosterone sulfate.**

| **Age** | **Women (µg/dL)** | **Men (µg/dL)** |
| --- | --- | --- |
| 1–7 days | 69–472 | 85–423 |
| 8–15 days | 33–350 | 30–176 |
| 16 days–3 years | <47 | <99 |
| 4–6 years | <47 | <228 |
| 7–8 years | 72–179 | 7–119 |
| 9–10 years | 12–195 | 14–89 |
| 11 years | 8–100 | 19–192 |
| 12 years | 24–266 | 41–371 |
| 13 years | 24–170 | 133–289 |
| 14 years | 22–327 | 144–300 |
| 15 years | 22–351 | 150–304 |
| 16 years | 56–385 | 210–365 |
| 17 years | 85–405 | 239–365 |
| 18–19 years | 65–380 | 280–440 |
| 20–29 years | 65–380 | 280–530 |
| 30–39 years | 45–270 | 120–520 |
| 40–49 years | 32–240 | 95–530 |
| 50–59 years | 26–200 | 70–310 |
| 60–69 years | 17–130 | 42–270 |
| ≥70 years | 17–130 | 42–195 |

**Table S3. Immunohistochemical marker expression in PAL patients.**

| **Patient number** | **CD3** | **CD5** | **CD10** | **CD20** | **CD30** | **MUM1** | **MYC** | **BCL2** | **BCL6** | **Hans phenotype** | **Double Expressor Status** |
| --- | --- | --- | --- | --- | --- | --- | --- | --- | --- | --- | --- |
| 1 | 0 | n/d | 0 | 1 | 1 | 1 | 1 | 1 | 1 | non-GCB | DE |
| 2 | 0 | 0 | 0 | 1 | 0 | n/d | n/d | 0 | 0 | non-GCB | c/d |
| 3 | 1 | n/d | n/d | 1 | n/d | 0 | 0 | 1 | 1 | c/d | nDE |
| 4 | 0 | 0 | 0 | 1 | nd | 1 | n/d | 1 | 1 | non-GCB | c/d |
| 5 | 0 | 0 | 1 | 1 | 0 | n/d | n/d | 1 | 1 | GCB | c/d |
| 6 | 0 | 1 | 0 | 1 | n/d | 1 | n/d | 1 | 1 | non-GCB | c/d |
| 7 | 0 | n/d | n/d | 1 | 0 | 1 | 1 | 1 | 1 | c/d | DE |
| 8 | 0 | n/d | 0 | 1 | 0 | 1 | 0 | 0 | 0 | non-GCB | nDE |
| 9 | 0 | 0 | n/d | 1 | 0 | 1 | 1 | 1 | 1 | c/d | DE |
| 10 | 0 | 1 | 0 | 1 | n/d | n/d | n/d | 1 | 1 | c/d | c/d |
| 11 | 0 | 0 | 0 | 1 | 0 | 0 | n/d | 1 | 1 | GCB | c/d |

0 – negative expression, 1 – possitve expression, non-GBC – no germinal center B-cell fenotype, GBC – germinal center B-cell fenotype, DE-double expressor MYC/BCL2, nDE- not double expressor MYC/BCL2, n/d – no data, c/d-cannot determine

**Table S4. Immunohistochemical marker testing and positivity rates in PAL cases**

| Marker | Tested (n) | Positive (n) | Positive (%) |
| --- | --- | --- | --- |
| CD3 | 11 | 1 | 9.1% |
| CD5 | 7 | 2 | 28.6% |
| CD10 | 8 | 1 | 12.5% |
| CD20 | 11 | 11 | 100.0% |
| CD30 | 7 | 0 | 0.0% |
| MUM1 | 8 | 6 | 75.0% |
| MYC | 5 | 3 | 60.0% |
| BCL2 | 11 | 10 | 90.9% |
| BCL6 | 11 | 9 | 81.8% |

**Table S5. All laboratory features of patients with PAL, ADE, PCC and ACC.**

| Parameter | PAL | ADE | PCC | ACC | p (4 gr.) | PAL vs ADE (p) | PAL vs PCC (p) | PAL vs ACC (p) |
| --- | --- | --- | --- | --- | --- | --- | --- | --- |
| n | 12 | 31 | 18 | 19 | - | - | - | - |
| Hemoglobin (g/dl) | 11.3 (±2.4) | 13.7 (±1.8) | 13.5 (±1.4) | 12.8 (±2.4) | 0.005 | 0.003 | 0.017 | 0.166 |
| MCV (fl) | 86.5 (±6.5) | 91.9 (±5.7) | 89.4 (±5.4) | 88 (±5.3) | 0.012 | 0.015 | - | - |
| MCH (pg) | 28.7 (±2.4) | 31.3 (±1.9) | 29.7 (±2) | 29.3 (±2.6) | 0.019 | 0.005 | 0.549 | 0.831 |
| RDW (%) | 86.2 (83.5-88.8) | 92.6 (89-94.7) | 88.3 (85-93.9) | 87.5 (83.9-90) | <0.001 | 0.013 | 0.105 | 1 |
| Leukocytes (×10⁹/l) | 7.1 (4.98) | 8.1 (3.1) | 7.41 (3.52) | 8.5 (3.84) | 0.696 | - | - | - |
| Neutrocytes (×10⁹/l) | 3.16 (2.41-5.57) | 4.98 (3.06-6.09) | 3.56 (2.91-4.55) | 4.89 (2.71-7.24) | 0.303 | - | - | - |
| Monocytes (×10⁹/l) | 0.9 (0.47-1.53) | 0.64 (0.47-0.74) | 0.52 (0.36-0.67) | 0.62 (0.54-1.12) | 0.08 | - | - | - |
| Eosinophils (×10⁹/l) | 0.08 (0.02-0.15) | 0.12 (0.08-0.17) | 0.11 (0.07-0.14) | 0.13 (0.09-0.25) | 0.285 | - | - | - |
| Lymphocytes (×10⁹/l) | 1.48 (±0.91) | 2.01 (±0.76) | 1.92 (±0.74) | 1.71 (±0.69) | 0.19 | - | - | - |
| Plateles (×10⁹/l) | 270 (224-297) | 247 (200-296) | 262 (221-297) | 301 (213-354) | 0.201 | - | - | - |
| MPV (fl) | 10.5 (9.9-11.1) | 11 (10.6-11.5) | 11 (10.5-11.5) | 10.2 (9.6-10.6) | <0.001 | 0.071 | 0.206 | 0.775 |
| Creatinine (mg/dl) | 1.05 (0.8-1.67) | 0.68 (0.6-0.8) | 0.71 (0.64-0.77) | 0.78 (0.6-0.97) | 0.029 | 0.014 | 0.034 | 0.233 |
| LDH****  (U/l) | 1. 37–280) n=12 | - n=0 | 162 (150.5-173.5) n=3 | 475 (208.5-703) n=7 | 0.058 | - | - | - |
| β2-microglobulin (mg/dl) | 5.85 (3.51–7.96) n=11 | 1.947 n=1 | 1.453 n=1 | 2.406 (1.74-2.5) n=3 | - | - | - | - |
| CRP (mg/l) | 71.89 (36.5–121) n=12 | 8.77 (2–10.7.) n=14 | 10.61 (1.79-14.3) n=11 | 63.77 (2.9-103.5) n=15 | <0.001 | 0.002 | 0.01 | 1 |
| ESR (mm/h) | 20 (14.25–34.75) n=10 | 2 n=1 | 21 (20-22) n=2 | 7 (4.5-9.5) n=2 | - | - | - | - |
| Ferritin (ng/ml) | 1274 (431-2021) n=7 | 147 (93-147) n=6 | - n=2 | 86 (68-103) n=3 | 0.003 | 0.01 | - | 0.005 |
| TAG (mg/dl) | 150 (97.5–172) n=8 | 137 (80.5–162) n=11 | 54 n=1 | 161 (161–169) n=5 | 0.235 | - | - | - |
| HDL cholesterol (mg/dl) | 27 (17.3–39.8) n=7 | 64 (35–65.5) n=11 | 86 (71.5–105.5) n=2 | 42 (28–42) n=5 | 0.179 | - | - | - |
| Albumin (g/l) | 34.5 (28–35.5) n=12 | 36 (36–36) n=1 | 38 (36–43) n=7 | 34.5 (25.5–39.5) n=12 | 0.087 | - | - | - |
| Corrected calcium (mg/dl) | 9.69 (9.32–10.4) n=12 | 9.5 (8.9–9.9) n=1 | 9.66 (9.4–9.8) n=7 | 9.42 (9.2–9.82) n=6 | 0.46 | - | - | - |
| Post DST cortisol (nmol/l) | 142 (41.5–293) n=6 | 97 (70–145) n=29 | 14 (113.8–89) n=4 | 90.5 (29.5–216.2) n=12 | 0.102 | - | - | - |
| Abnormal DST (%) | 4 (66.7)n=6 | 23 (79.3) n=29 | 2 (66.6) n=4 | 7 (58.3) n=12 | 0.014 | 0.722 | 0.37 | 1 |
| DHEA-S*** (µmol/l) | 73.1 (7.5–109) n=9 | 21.4 (7.5–70.1) n=31 | 87.1 (37.2–138) n=18 | 187.3 (76.3–313.2) n=16 | <0.001 | 0.843 | 1 | 0.078 |
| DHEA-S > ULN n (%)*** | 0 (0) n=9 | 1 (3.2) n=13 | 6 (31.6) n=18 | 8 (50) n=16 | <0.001 | 1 | 1 | 0.136 |
| Metanephrines ULN % *** | 30% (12–67) n=7 | 41% (27–60.5) n=27 | 327.4 (92–535) n=17 | 64.5 (39–77) n=16 | <0.001 | 1 | 0.002 | 0.704 |
| Normetanephrines ULN %*** | 89 (40–104) n=7 | 78 (49–111) n=27 | 219 (147–666) n=17 | 79.5 (67.5–92.5) n=16 | <0.001 | 1 | 0.011 | 1 |
| % patients with metanephrine/normetanephrine > 2× ULN | 14.3% n=7 | 3.7% n=27 | 88.2% n=17 | 0 n=16 | <0.001 | 0.374 | <0.001 | 0.304 |

*The number of individuals in the group (n) is provided only for outcomes that were not assessed in all participants within the group. The absence of group size information indicates that the assessment was performed for all participants. MCV- mean corpuscular volume, MCH- mean corpuscular hemoglobin, RDW- red blood cell distribution width, MPV-mean platelet volume, LDH-lactate dehydrogenase, CRP-c-reactive protein, ESR-erythrocyte sedimentation rate, TAG- triglycerides, HDL- high-density lipoprotein, DST- dexamethasone suppression test, ULN- upper limit of normal, **Statistical analysis was performed only between two groups due to the insufficient number of examination results in the other groups,***Normal range depended on patient’s age, sex and the year of examination.*
